# Adherence to a care bundle for *Staphylococcus aureus* bacteraemia: A retrospective cohort study

**DOI:** 10.4102/sajid.v37i1.445

**Published:** 2022-11-22

**Authors:** Elizabeth M. Gatley, Tom Boyles, Sipho Dlamini, Marc Mendelson, Phiona E. Namale, Peter J. Raubenheimer, Sean Wasserman

**Affiliations:** 1 Department of Medicine, Faculty of Health Sciences, Groote Schuur Hospital, University of Cape Town, Cape Town, South Africa; 2 Department of Medicine, Faculty of Health Sciences, Division of Infectious Diseases and HIV Medicine, Groote Schuur Hospital, University of Cape Town, Cape Town, South Africa

**Keywords:** *Staphylococcus aureus* bacteraemia, bloodstream infections, infectious disease consultation, bundle of care, adherence

## Abstract

**Background:**

*Staphylococcus aureus* bacteraemia is associated with high hospital mortality. Improvements in outcome have been described with standardised bundles of care.

**Objectives:**

To study the adherence of a standardised bundle of care (BOC) recommendations using a consultation pro forma, for all patients admitted with *S. aureus* bacteraemia to Groote Schuur Hospital over a year. The study further aimed to describe the 90-day mortality in these patients and to assess for an association between adherence to the bundle of care and outcome.

**Method:**

A retrospective audit of all unsolicited infectious disease consultations for patients with *S. aureus* bacteraemia admitted to Groote Schuur Hospital during 2018. Adherence to recommendations of a standard bundle of care was audited.

**Results:**

A total of 86 patients were included in the study: 61 (71%) with hospital-associated infection and 25 (29%) with community-associated infection. Over 80% of adherence to treatment recommendations was achieved regarding antibiotic (including vancomycin) usage, source control and use of echocardiography as required. In-hospital mortality was 16%, while the overall 90-day mortality was 18%, with only age as an independent predictor of mortality. No association between adherence to the bundle of care and outcome was found.

**Conclusion:**

Adherence to a simple, structured bundle of care was good when using standardised pro forma as communication tools for advice and a structured antibiotic chart for vancomycin administration. Although adherence was not associated with outcome, the overall mortality for *S. aureus* bacteraemia was improving in the institution under study.

**Contribution:**

Our findings support feasibility and ongoing use of bundles of care for *S. aureus* bacteraemia in similar settings.

## Introduction

*Staphylococcus aureus* bacteraemia (SAB) remains one of the most common causes of community- and healthcare-associated blood stream infections (BSI) globally. It is associated with a significant burden of morbidity and mortality, and increased healthcare costs.^1,2,3,4^ To improve outcomes in SAB, greater emphasis has been placed on the benefit of infectious disease consultation (IDC), as well as on a bundle of care (BOC) package to guide the treatment of SAB.^5,6,7,8,9^

In 2013 the Division of Infectious Diseases (ID) at Groote Schuur Hospital (GSH) started a process of reviewing all cases of SAB admitted to the hospital. Outcomes of the first 100 cases have been published, showing a high mortality of 47%.^10^ That survey specifically identified sub-optimal antibiotic usage and poor adherence to recommendations for source control as possible contributors to mortality. On 01 September 2015 the Division expanded their scope to provide unsolicited IDC on all cases of SAB using a newly created BOC. This BOC was drawn up by a panel of ID specialists at GSH and agreed upon in accordance with best practice guidelines described in a 2011 *Lancet Infectious Diseases* review^11^ and a 2014 *Journal of the American Medical Association (JAMA)* review on the management of SAB.^12^ It included an agreed-to standardised approach to treatment and a standardised pro forma to improve communication regarding recommendations to the treating team (Appendices). The focus was to ensure adherence to key quality of care indicators (QCIs), including correct definitive antibiotic use with dose, dosing interval and length of treatment, and in the case of vancomycin use, correct initial loading dose and use of therapeutic drug monitoring required to adjust dosing. Further QCIs included early source control, performance of 72 h blood cultures and appropriate use of echocardiography in specific clinical scenarios.

In this study, the authors aimed to retrospectively evaluate adherence to the specific QCIs and review the impact on patient mortality.

## Research methods and design

### Study setting and population

The data for this study were collected from patients admitted to the hospital, which serves as a secondary level hospital for its local drainage area and a tertiary referral centre for the entire Cape Town metro west area.

### Recruitment and enrolment

The researchers retrospectively analysed records of all patients with SAB seen by the ID Division in 2018, following implementation of the new, routine, BOC (Appendix 1). The care bundle consisted of the following: all blood cultures for admitted patients that are positive for *S. aureus* are routinely reported to clinicians in the Division of Infectious Diseases and HIV Medicine, who provided an unsolicited consultation to the treating team within 24 h or the first day after a weekend. Advice on further management of each patient was conveyed to the managing team via a pro forma that was stapled into the patient’s confidential file with clear recommendations, including the need for echocardiography, source control, recommendations with regard to antibiotic choice, dose, dosing interval and length of treatment (Appendix 2). When relevant, this included a dedicated vancomycin prescription chart, which provided information on vancomycin dosing and therapeutic drug monitoring (Appendix 3). All patients with SAB were seen weekly by the ID team, or more frequently if required, until their discharge from hospital.

### Inclusion criteria

Patients were included in the study if they were aged > 18 years, and *S. aureus* was isolated from one or more blood culture regardless of clinical signs of systemic infection.

### Exclusion criteria

Patients in whom *S. aureus* was isolated as part of a mixed growth of organisms were excluded, as were patients who died within 48 h of admission and thus were not subject to the intervention, and those in whom the therapeutic strategy was one of palliation rather than active intervention.

### Definitions

Community-associated SAB (CA-SAB) was defined as a blood culture positive for *S. aureus* taken on or within 48 h of admission. Hospital-associated SAB (HA-SAB) was defined as a blood culture positive for *S. aureus* taken after 48 h following admission or in a patient with a positive blood culture within 48 h of admission who had (1) received intravenous antibiotics in the preceding 30 days, (2) attended hospital or received haemodialysis in the previous 30 days or (3) a patient who resides in a long-term care facility. Definite line sepsis was defined as active drip site sepsis in the preceding 30 days based on a clinical diagnosis. Probable line infection was considered in those with an HA-SAB with current or recent (< 30 days) intravenous line and no other obvious focus of infection. Death was considered SAB-related if there were persistent signs and symptoms of BSI or a persistently positive blood culture at the time of death. Optimal definitive antibiotics include correct dose, dosing interval and length of treatment, and consisted of the following: methicillin-susceptible *S. aureus* (MSSA) bacteraemia was treated with either intravenous cefazolin or cloxacillin depending on what was available in pharmacy and whether there was evidence of endocarditis, meningitis or osteomyelitis. Cefazolin 2 g 8 hourly or cloxacillin 2 g 6 hourly was considered an acceptable treatment for MSSA bacteraemia unless the patient was deemed to have infective endocarditis or osteomyelitis, where the recommendation was cloxacillin 3 g 6 hourly. Meningitis in a patient with MSSA bacteraemia was treated with ceftriaxone 2 g 12 hourly in view of unclear evidence of adequate absorption of cloxacillin into the blood brain barrier.^13^ Methicillin-resistant *S. aureus* (MRSA) bacteraemia was treated with vancomycin. Patients required an initial loading dose of 25 mg/kg – 35 mg/kg followed by 10 mg/kg – 15 mg/kg 12 hourly. Ongoing dosing was dependent on therapeutic drug monitoring. A vancomycin dosing and monitoring chart was stapled into the patient’s treatment chart. Length of treatment was based on local and international accepted guidelines.^14^ Uncomplicated SAB was treated for 14 days. Complicated infection was defined as (1) persistently positive blood culture > 72 h after initiating treatment with an appropriate definitive antibiotic and/or failure of clinical resolution of clinical features of infection; (2) infective endocarditis; or (3) evidence of metastatic infection, for example, discitis. These patients required treatment for at least 28 days. The 72-h blood culture was considered correctly performed if it was taken within 48 h – 96 h following initiation of antibiotics that the cultured *S. aureus* was susceptible to.

### Data management

All data were anonymously collected into a RedCap^®^ database. Only members of the research team and ID Division had access to records to maintain patient confidentiality. All data management programmes were password protected.

### Statistical analysis

Data management and statistical analysis were conducted in SPSS Statistics Version 28.0 (SPSS Inc., Chicago, Ill., United States). Descriptive statistics were used to summarise patient data, namely, frequency and percentage for categorical data and mean with standard deviation (s.d.) for continuous data. Kaplan-–Meier estimates were used to generate cumulative risks and the Cox proportional hazards model was used to determine the effect of the intervention on the outcome and to estimate hazard ratios (HR) and 95% confidence intervals (CI). Variables included in the Cox model were selected based on best evidence. For all statistical tests, a *p*-value of 0.05 was considered significant.

### Ethical considerations

This study was approved by the Human Research and Ethics Committee at the University of Cape Town (Ref No. 766/2018).

## Results

There were 119 SAB infections that were subject to the BOC intervention during 2018 of which 86 cases were included in this analysis. Cases were excluded for the following reasons: incomplete data 0 (17); age < 18^3^ multiple organisms on blood culture^5^ and palliative care only provided.^8^

### Patient and infection characteristics

A total of 57 (66%) patients were men and the mean age was 46.1 years (s.d.: 16.3 years). Patients had a wide range of comorbidities, the most common being hypertension, diabetes mellitus, chronic kidney disease and underlying malignancy. Nine patients were HIV-positive, with eight on anti-retroviral medication (ART). The median CD4 T-cell count was 400 cells/mm^3^ (interquartile range [IQR]: 146–513) (Table 1).

**TABLE 1:** Characteristics of patients admitted to the hospital with *Staphylococcus aureus* bacteraemia over the 12-month period.

Variables	Healthcare-associated (*n* = 61)		Community-acquired (*n* = 25)		Total (*n* = 86)	
	*n*	%	*n*	%	*n*	%
**Age**						
> 60 years	15	25	5	20	20	23
**Male gender**	41	67	16	64	57	66
**Comorbidities**						
Any	41	67	20	80	61	72
Hypertension	16	26	4	16	20	23
Diabetes mellitus	13	21	6	24	19	22
Chronic kidney disease	9	15	2	8	11	13
Chronic respiratory disease	3	5	1	4	4	5
Stroke	2	3	4	16	6	7
Seizure disorder	4	7	2	8	6	7
Other neurological conditions	4	7	3	12	7	8
Cardiological condition	7	12	1	4	8	9
Urological condition	4	7	2	8	6	7
Dermatologic condition	3	5	2	4	5	6
HIV	3	5	6	24	9	11
Malignancy	9	15	3	12	12	14
**Type of SAB**						
MRSA	11	18	0	-	11	13
MSSA	50	82	25	100*	75	87
**Source of infection**						
Line-related infections (all)	33	54	0	-	33	38
Peripheral line (definite)	12	20	0	-	12	14
Peripheral line (probable)	10	16	0	-	10	12
Central line	6	10	0	-	6	7
Dialysis catheter	5	8	0	-	5	6
SSTI	11	18	14	56*	25	29
Pneumonia	2	3	2	8	4	5
Surgical site	7	11	0	-	7	8
Intravenous drug use	0	-	1	4	1	1
Other	3	5	3	12	6	7

Note: Age in years. Healthcare-associated: mean = 46.0, s.d. = 16.8; Community-acquired: mean = 46.4 s.d. = 15.1; Total: mean = 46.1 s.d. = 16.3.

HIV, human immunodeficiency virus; MRSA, methicillin-resistant *Staphylococcus aureus*; MSSA, methicillin-sensitive *Staphylococcus aureus*, HCA-SAB, healthcare-associated *Staphylococcus aureus* bacteraemia; CA-SAB, community-acquired *Staphylococcus aureus bacteraemia*; SSTI, skin and soft tissue infection.

* , No characteristic differed significantly between the study groups (*p* = 0.05 at baseline according to Fisher’s exact test for categorical data or the Wilcoxon rank-sum test for continuous data), with the exception of type of SAB (*p* = 0.029) and the presence of skin and soft tissue infections (*p* = 0.001).

A total of 25 patients (29%) were admitted with CA-SAB and 61 (71%) with HA-SAB. All 25 cases of CA-SAB were MSSA. Of the HA-SAB, 11 (18%) were MRSA and 50 (82%) were MSSA. Peripheral or central line-associated BSI accounted for 33 (53%) of the HA-SAB episodes: ‘definite peripheral line infection’ in 12 patients (20%), ‘probable peripheral line infection’ in 10 (16%) patients, dialysis catheter-related infection in five patients (8%) and other central line-related infections in six patients (10%). Skin and soft tissue infection was the primary source of infection in 25 (29%) patients, and was more common in patients with CA-SAB.

### Adherence to quality of care indicators

Adherence to the five QCIs indicators are listed in Table 2. Overall adherence of 100% was only achieved in 45 cases and less than 50% adherence was seen in 19 patients. Adherence to the five separate QCIs comprising the SAB BOC was > 80% in four out of the five QCIs, with the 72-h blood culture performing worst, having taken place at the correct time in only 56 (65%) cases. Advice given on source control was required in 40 cases, of which it was correctly adhered to in 34 (85%) cases. Echocardiography was recommended in 31 cases (36%) and was performed in 26 (83.8%) cases, resulting in the identification of three patients (11.6%) with evidence of infective endocarditis and vegetations visible on at least one valve. Thirteen cases were not adequately treated with antibiotics – eight (62%) were based on too short a course of intravenous antibiotics while the remainder were deemed inadequate because of sub-therapeutic vancomycin levels or incorrect dosing of alternate antibiotics. Of the 11 patients treated with vancomycin, 10 (90%) received a loading dose. Therapeutic drug monitoring with trough levels was performed at least once in all 11 patients but only recorded to be therapeutic in nine (81%). Nine out of the 11 patients (81%) had both a loading dose and therapeutic vancomycin levels recorded.

**TABLE 2:** Adherence to quality care indicators.

Component	Adherence		
	*n*	Required	%
72-h blood culture	56	86	65.1
Source control	34	40	85.0
ECHO	26	31	83.9
Vancomycin administration	9	11	81.8
Antibiotic appropriateness	73	86	84.9

Note: Source control, appropriate source control required; ECHO, echocardiography performed as recommended; Vancomycin administration, correct loading dose and follow-on doses guided by serum level monitoring; antibiotic appropriateness, correct choice of empiric and definitive antibiotic choice.

### Outcomes

Inpatient mortality was 16% (14 patients): 11 (78%) deaths were attributable to SAB and the remaining three were considered unrelated. Two further patients died following discharge from hospital, thus increasing the 90-day mortality to 18%. Neither of the two outpatient deaths was considered SAB related. Of the 11 deaths secondary to SAB, there was one MRSA and 10 MSSA BSIs. Six patients had CA-SAB and five had HA-SAB. Patient and infection characteristics for the cohort as a whole, stratified by 90-day outcome, are shown in Table 3. Patients who died were significantly older, and more likely to be hypertensive or have a stroke.

**TABLE 3:** Patients and clinical characteristics of patients who survived compared to those that died with *Staphylococcus aureus* bacteraemia.

Variables	Alive (*n* = 70)		Dead (*n* = 16)	
	*n*	%	*n*	%
**Age**				
> 60 years	10	14	10	63*
**Male gender**	48	69	9	56
**Comorbidities**				
Any	46	66	15	94*
Hypertension	13	19	7	44*
Diabetes mellitus	13	19	6	38
Chronic kidney disease	7	10	4	25
Chronic respiratory disease	4	6	0	-
Stroke	2	3	4	25*
Seizure disorder	5	7	1	6
Other neurological condition	4	6	3	19
Cardiological condition	6	9	2	13
Urological condition	5	7	1	7
Dermatologic condition	3	4	2	13
HIV	7	10	2	12
Malignancy	8	11	4	25
**Type of SAB**				
MRSA	9	13	2	13
MSSA	61	87	14	88
HA-SAB	53	76	8	50
CA-SAB	17	24	8	50
**Source of infection**				
Line-related infections (all)	28	40	5	31
Peripheral line (definite)	9	13	3	19
Peripheral line (probable)	9	13	1	6
Central line	5	7	1	6
Dialysis catheter	5	7	0	-
SSTI	20	29	5	31
Pneumonia	3	4	1	6
Surgical site	6	9	1	6
UTI	6	8	0	-
Intravenous drug use	1	1	0	-
Other	4	6	2	13

Note: Age in years. Alive: mean = 42.8, s.d. = 14.9; dead: mean = 60.5, s.d. = 14.4).*

HIV, human immunodeficiency virus; MRSA, methicillin-resistant *Staphylococcus aureus*; MSSA, methicillin-sensitive *Staphylococcus aureus*, HCA-SAB, healthcare-associated *Staphylococcus aureus* bacteraemia; CA-SAB, community-acquired *Staphylococcus aureus* bacteraemia; SSTI, skin and soft tissue infection.

* , No characteristic differed significantly between the study groups (*p* = 0.05 at baseline according to Fisher’s exact test for categorical data or the Wilcoxon rank-sum test for continuous data), with the exception of age > 60 (*p* < 0.001) and the presence of hypertension (*p* = 0.048) and stroke (*p* = 0.01).

**FIGURE 1:**
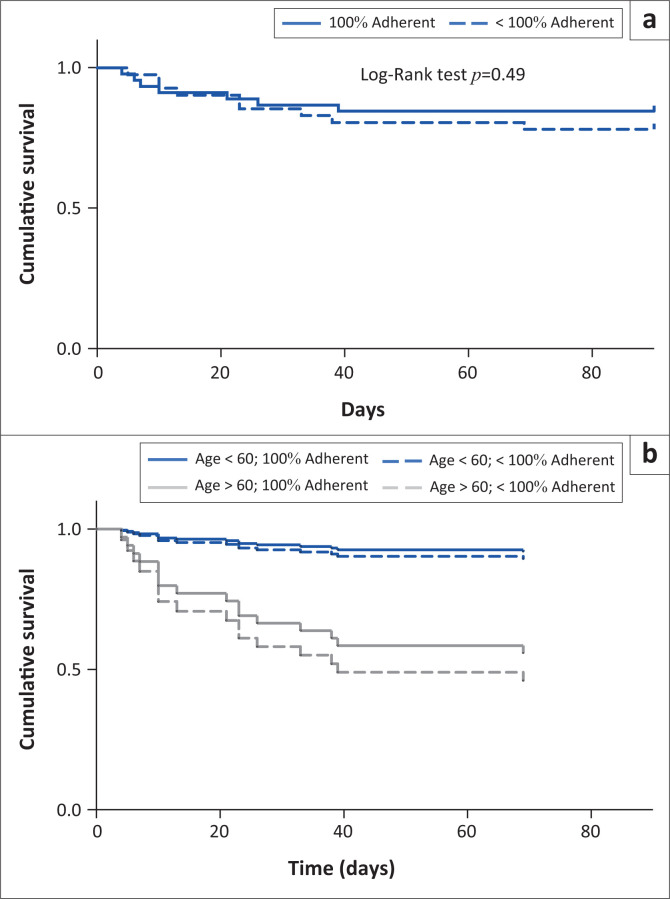
Survival curves for patients with *Staphylococcus aureus* bacteraemia. (a) Kaplan–Meier curve for patients with full versus < 100% adherence. (b) The Cox proportional hazards regression stratified by age and adherence. Hazard ratio (HR) (95% confidence interval [CI]) for age > 60 versus not: 7.0 (2.6–20.5); HR (95% CI) for 100% adherent versus not 0.8 (0.3–2.0).

There was no association between adherence to the SAB bundle and survival, both with adherence expressed as a continuous variable or as a categorical ‘100% adherence’ versus not. The unadjusted survival estimates for those with 100% adherence versus those without are shown in Figure 1a, and with age as an interaction term in Figure 1b. When examined individually, only the absence of ‘source control’ was shown to be significantly associated with mortality; absent in 65% of those who died versus 12% of those who did not; HR 8.814 (2.149–36.153, *p* = 0.003). Univariable predictors of death in the Cox regression included age and community-acquired infection; on multivariable analysis, only age was an independent predictor of outcome (Table 4). There was no difference in overall adherence to the BOC between those over the age of 60 or under, in those with MSSA versus MRSA, or in those with CA-SAB or HA-SAB.

**TABLE 4:** Cox regression analysis to predict the 90-day mortality in patients with *Staphylococcus aureus* bacteraemia.

Parameter	Univariate		*p*	Multivariable		*p*
	HR	95% CI		HR	95% CI	
Age (per additional year)	1.07	1.035–1.107	< 0.001	1.071	1.033–1.111	< 0.001
Male gender	0.654	0.243–1.755	0.399	-	-	NS
Comorbidity (any)	6.659	0.879–50.427	0.066	-	-	NS
MRSA	0.876	0.199–3.855	0.861	-	-	NS
Community-acquired infection	2.712	1.017–7.234	0.046	-	-	NS
Full adherence to bundle	0.726	0.270–1.949	0.525	-	-	NS

HR, hazard ratio; CI, confidence interval; MRSA, methicillin-resistant *Staphylococcus aureus;* NS, not significant.

## Discussion

This study was able to demonstrate that with a clear and structured BOC intervention, adherence to key QCIs for the treatment of SAB was possible, even in a low-resource setting such as South Africa. Four out of the five identified QCIs achieved adherence rates of greater than 80%. While similar interventions have been introduced in multiple hospitals worldwide, this is the first study to the authors’ knowledge, evaluating a BOC for the treatment of SAB both within South Africa and on the African continent. Overall inpatient mortality for this study population was approximating that of best international practice at 16%. However, the study did not find that better adherence to the QCIs was associated with improved survival. Age, as published elsewhere, was the strongest predictor of a poor outcome.^4,6^

There is already a significant body of evidence that shows improved patient outcomes when an ID specialist is involved in the management of patients with SAB.^6,7,8,9^ This evidence is so compelling that Tong et al. suggested that it should be ‘the standard of care where ID physicians are available’.^15^ The reason for improved outcome is likely aggressive application of standard of care treatment guidelines including early localisation of infection, source control, use of ECHO and early initiation of appropriate antibiotic therapy. This has led to a focus on a BOC for the treatment of SAB and the impact it may have on patient outcomes.

Lopez-Cortes et al. detailed the introduction of a BOC across 12 tertiary hospitals in Spain.^8^ The structured bundle showed an increased adherence to six evidence-based QCIs they had identified through the literature and was associated with a reduction in mortality. In 2014, a small 200-bed community hospital in Germany was able to show a significant reduction in inpatient mortality from 44% to 10% with the introduction of a structured BOC for SAB.^16^ Infectious diseases specialists were available at the hospital and consulted on all SAB cases following the intervention. In 2016, Townsend et al. showed a significant improvement in adherence to standard guidelines for the treatment of SAB and a decrease in the 90-day relapse rate with a trainee-initiated and run intervention in a large public hospital serving many uninsured patients with significant comorbidities in Texas.^17^ This was achieved by developing an institutional protocol for the treatment of SAB which was distributed to healthcare providers in multiple formats. The intervention was entirely trainee-run, a crucial step towards greater care for more patients with SAB and not just those in large institutions with access to IDC.

In 2017, a study from Japan evaluated patients with SAB who survived 14 days in hospital and assessed adherence to QCIs and the effect on mortality.^18^ An increase in adherence to at least four out of five QCIs rose from 47.5% to 79.3%, with a decreasing mortality from 10.0% to 3.4% over an 8-year period. In 2018, a similar mortality benefit was seen with a BOC for catheter-related infections because of MSSA. In this cohort, adherence of > 55% to the BOC already showed an improvement in 30-day all-cause mortality.^19^ In 2020, an evaluation of outcomes in a New Zealand tertiary hospital following the introduction of a BOC for treatment of SAB again showed a significant impact on patient outcomes.^20^ While 30-day mortality rates were not significantly different in the two arms, relapse rates and thus morbidity significantly decreased in the post-intervention arm with increased adherence to treatment guidelines.

Early studies from South Africa described the epidemiology and risk factors associated with SAB and poorer outcomes.^21,22,23^ A previous paper from this institution, describing a sequential 100 admissions with SAB, found a 90-day mortality rate of 47%, and described problems with overall care.^10^ Prescription of definitive antibiotics was inadequate in a quarter of all study participants and up to one-third of those with MRSA. Early source control was poorly adhered to; < 60% of those requiring source control received it. After introduction of the BOC, this article describes an improvement in ideal antibiotic usage and adherence to recommendations regarding source control to 80%, similar to that reported in the mentioned international studies. The mortality rate in the present study was lower than in most observational studies of SAB, partly because the present authors excluded patients who died within 48 h of admission or in whom curative therapy was not attempted. Direct comparison with the previous audit from this institution was not possible because of the different enrolment criteria.

A major strength of this study was the ability to accurately capture and evaluate all episodes of SAB in a single hospital. A simple tick sheet was easy to implement and to use for standardising recommendations to treating clinicians. There are several important limitations, including the small sample size and its retrospective nature, both of which may introduce bias and limit statistical analysis. The authors could not assess why there was a lack of association between BOC and outcomes as a whole or of the individual components of the BOC. It might be that the overall adherence was good enough (with the exception of the 72 h blood culture – which may be less important) that such an association could not be shown. Alternatively, institutional culture of caring for these infections may have changed over time with awareness raised because of the BOC roll-out such that overall care also improved. Patients not subject to the BOC intervention were not included in this analysis and thus a true inpatient mortality rate is unclear. Vancomycin dosing, since the start of this study, has changed and is now based on area under the curve (AUC)-dosing rather than trough dosing.^24^ This BOC included consultation by ID specialists, which cannot be generalised to hospitals without such support.

## Conclusion

This study shows that a BOC for SAB in-hospital that includes routine, unsolicited ID consultation with a pro forma for recommendations and a vancomycin loading dose chart resulted in good adherence to standard guideline recommendations and a good outcome for patients in the present study setting. Future studies should attempt to prospectively investigate the impact of a similar BOC intervention for SAB in hospitals without ID specialist support.

## Appendix 1: Unedited copy of the Infectious Diseases and Microbiology consensus document for treatment of *Staphylococcus aureus* bacteraemia as used during the bundle of care service provision

It is intended that all patients at the hospital with *Staphylococcus aureus* bacteraemia (SAB) are seen by an infectious disease consultant at least once either as a solicited or as an unsolicited consultation. This document seeks to clarify a unified approach to investigating and managing patients with SAB.

Definitions

All patients >13 years old with a single blood culture growing *S. aureus* are considered to have SAB. Definitions are consistent across a number of publications stating that low-risk patients have ALL of the following:

- nosocomial acquisition
- sterile blood cultures approximately 72 h after stating effective therapy
- no permanent intracardiac device or vascular graft material
- are not dialysis dependent
- no peripheral signs of endocarditis
- no cardiac murmur
- no clinical signs of metastatic foci of infection.

Investigations

In line with multiple guidelines and publications from respected authorities, all patients should undergo a repeat blood culture after 72 h of antibiotic therapy and if positive repeat cultures every 72 h until negative. They should also have a chest X-ray and focused imaging to determine the foci of infection, depending on clinical features.

Echocardiography

- All patients with prosthetic heart valves or implantable cardiac devices should be referred to cardiology for opinion on the need for transoesophageal echocardiograph (TEE) unless (all below are met):
  ■ There is an obvious treatable source.
  ■ There is resolution of bacteraemia and sepsis syndrome within 72 h.
  ■ The clinical suspicion (signs) of Infective endocarditis (IE) is low.
  Patients defined as low risk do not require routine transthoracic echocardiography (TTE).
  All patients **not** defined as low risk should have a TTE (this includes all patients with a murmur).
  Other than as stated above, TEE is reserved for patients in whom TTE is technically difficult and adequate views cannot be obtained, for example, obesity, ICU patients.
  Repeat TTE after 2 weeks of treatment may be appropriate in certain cases where endocarditis is still considered.
  Infectious diseases may discuss other indications for TEE with cardiology on a case-by-case basis.

Initial antibiotics

Initial therapy for methicillin-sensitive *Staphylococcus aureus* (MSSA) is cloxacillin 2 g intravenous (i.v.) 6 hourly increased to 3 g for endocarditis, meningitis and osteomyelitis. Penicillin allergic patients should have vancomycin in the first instance. Cefazolin can be considered in patient with a non-type 1 hypersensitivity to penicillin. Referral to clinical immunology may be appropriate. Initial therapy for methicillin-resistant *Staphylococcus aureus* (MRSA) is vancomycin including a loading dose. Patients with prosthetic heart valves or an implantable cardiac device should be referred immediately to cardiology and are likely to require TEE. Rifampicin 300 mg 12 hourly and gentamicin 3 mg/kg 8 hourly should be added to standard therapy.

Source control

All intravascular lines should be removed or replaced immediately. Surgical source control should be performed at the earliest opportunity.

Duration of therapy

The Infectious Diseases Society of America (IDSA) definition of uncomplicated SAB when all the criteria below are met:

- Endocarditis has been excluded.
- There are no implanted prostheses.
- Follow-up blood cultures performed on specimens obtained 2–4 days after the initial set do not grow *S. aureus.*
- Defervescence of fever occurs within 72 h of initiating effective therapy.
- No evidence of metastatic infection is present on examination.

All patients with uncomplicated SAB should have 14 days i.v. therapy from the date of first negative blood culture.^1^ Patients with complicated SAB should be treated for 4–6 weeks. These cases can be discussed on an individual basis between ID and micro when a switch to oral therapy may be considered in selected cases. N.B. Being at high risk does not necessarily mean having complicated SAB. For example, patients with community acquired infection or cardiac murmurs can be treated as uncomplicated if they fulfil the criteria above. Guy Thwaites’ LID paper^2^ discusses the lack of evidence for switching to oral therapy after initial intravenous therapy although there is some observational data suggesting that it may be safe in complicated SAB after at least 2 weeks of intravenous therapy. Generally linezolid is not recommended for the treatment of SAB. However, it has been used successfully as an oral option following vancomycin in patients with *S. aureus* infections. There is a lack of direct evidence in uncomplicated MRSA bacteraemia but it may be reasonable to switch patients with who have a good clinical response to vancomycin to linezolid to facilitate hospital discharge.

Follow-up

All patients will be seen weekly until discharge or more frequently if required.

## Appendix 2: Unsolicited infectious diseases consultations to improve outcomes of patients with *Staphylococcus aureus* bacteraemia

In line with International best practice the Infectious Diseases service is providing unsolicited evidence-based recommendations for the management of all patients with *Staphylococcus aureus* bacteraemia (SAB).
